# Sanatorium Experiences

**Published:** 1907-11-23

**Authors:** E. J. Sunnucks


### The General Practitioner's Column.

[Contributions to this Column are invited, and if accepted will be paid for.]

### SANATORIUM EXPERIENCES.

By E. J. SUNNUCKS, M.R.C.S.

Within late years we have heard so much about sanatoria for the cure of pulmonary tuberculosis, that a few personal experiences in a well-known sanatorium in Davos under German control may not prove uninteresting. Perhaps it would be as well before going further to outline briefly the daily routine of life there. About 6.30 a.m. an attendant comes to my room to give me "Abreibung." This means the application of a wet cloth all over the body, and afterwards drying with a rough huckaback towel in a vigorous manner. Although this is very agreeable in the summer, it has its drawbacks in the winter, when at 6.15 a.m. I am awakened from a sound sleep by a knock at my door. Of course it is pitch dark, and the thermometer is well below freezing-point. I call out "herein," and forthwith a burly figure enters, switches on the electric light, and closes the window. After that he proceeds to break the ice in my water jug, and pouring out a little water in a basin soaks a small towel in the same. This he applies to my chest, a proceeding which effectually disposes of any sleepy feelings which may have remained after the first awakening. Then he takes the rough towel and rubs vigorously. In this way also the back and limbs are treated. The whole affair lasts about ten minutes. After switching off the light, I settle down to sleep again.

About 7.30 I am again awakened by the Zimmermädchen who has brought me hot water. This, of course, means getting up. The first breakfast is served from 7 to 8.30 in summer and from 7.30 to 9 in winter; if you are not down in time you get no breakfast. After the first breakfast I make "Liegekur" until 10.30 a.m. Liegekur—namely, lying-cure—consists of lying on a Liegestuhl or long invalid chair on a covered terrace, more or less wrapped up according to the time of year. When my condition had undergone improvement I was allowed to take a walk after the first breakfast, but of course the walks are restricted, and I was told to walk along a gradually inclined path as far as a certain seat, and then to return for more Liegekur. The second breakfast, served at 10.30, consists of milk and bread and butter. During this meal the doctor comes round, examines the temperature chart of each patient, and gives instructions. After this meal I am allowed to walk a prescribed time if the temperature is normal, but if not, then I again make Liegekur until 1 o'clock, when the gong for Mittagessen is sounded. This is the principal meal of the day, and consists of five courses. Temperatures are taken immediately after the meal is finished, and then Liegekur again from 2.30 till 4. During this time absolute rest is enjoined, even conversation being prohibited. At a quarter to 4 the doctors visit the patients on the Liegehalle. At 4 o'clock milk and coffee with biscuits, etc., are served in the Speisesaal, and afterwards patients go for their prescribed walks. Cure is again made from 6 to 7 p.m. At 7 p.m. the second great meal of the day, "Abendessen," is served; it consists of four courses. Then from 8 until 9 p.m. Liegekur is again made. At 9 p.m. the servants come round to the Liegehallen with hot milk. All patients must be in bed by 10 p.m. In this sanatorium, when I first arrived, there were many things to be learnt which were not always congenial to my inclinations. For instance, when in England I had been accustomed from childhood to have a cold bath every morning. The morning after my arrival I donned my dressing gown and made tracks for the bathroom. To my surprise I found it locked, and on expostulating with an attendant was told that no baths were allowed without the doctor's orders.

Smoking is not allowed unless the throat is quite in normal condition; all those allowed to do so smoke cigarettes except myself, and I invariably smoke a pipe. The food here is essentially different from our English fare. Most of the courses are prepared *à la française*, though now and then we get German dishes, such, for example, as pork and sauerkraut and Frankfurt sausages. I suppose one becomes accustomed to the diet in course of time, but I often think wistfully of the meals I had in England. Some time ago I got to know that we were going to have lamb and green peas for one of the courses at Mittagessen; knowing from experience that mint or mint sauce is never served with this dish, I ordered some of the latter to be prepared. When the course was served and I helped myself liberally to what I thought was mint sauce, imagine my disgust when I found that peppermint had been used in its preparation!

The doctors are generally three in number, and four in winter, when the sanatorium is filled to its utmost capacity. The chief doctor or "leitender Arzt" is a very genial man of some 50 years of age. He, as well as the other physicians, has had pulmonary tuberculosis, but some 20 years ago. He is regarded with awe by both patients and staff. His word is final, and complaints of all kinds, no matter how trivial, are all submitted to him. Although there is a managing director and also a housekeeper, both of them very capable people, yet the head doctor occupies himself with household, financial, and even culinary matters, for the menus for the day's meals must every morning early be submitted to his inspection. Under his strict rule no one is allowed to change his seat at table or in the Liegehalle without permission. It is quite surprising the number of people here who are expelled for transgression of the rules. For instance, one young fellow was sent away because he had been found smoking a cigarette in his room, another because he was discovered drinking champagne with a friend in his room after 10 p.m., but the most frequent cause of dismissal is partiality towards one of the other sex. Love-making, flirting, etc., are reckoned as heinous crimes, and are always followed by summary expulsion of both parties.

The doctors are all Germans, and they work very hard, for with an average of 70 patients there is plenty for them to do. There are always nine or ten patients in bed for various reasons, and in February or March influenza seems to be very prevalent every year, and often as many as 20 patients are then in bed with fever. The life of a doctor here is not very enjoyable, for there are rarely opportunities for dining out, theatres, card parties or such like, and the work is distinctly arduous.

One of the most remarkable things here, contrasting so strongly with the custom in English sanatoria, is the consumption of alcohol. Wines, liqueurs, etc., are allowed to a surprising extent, and more than one patient has received his *congé* on account of insobriety. I remember on one occasion a Swedish lieutenant had too much champagne one evening, and, smoking a cigar in his room, omitted to extinguish it before going to bed. In a short time his room was in flames, and it was only by the prompt action of the fire brigade and attendants that the fire was extinguished. Since that event the doctors keep a stricter eye on the amount of alcohol consumed. This consumption of alcohol is the same in nearly all German sanatoria. During the year certain feasts are kept called Festtage. On these occasions we have a Bier-Abend, when beer is free, and patients sit in the Speisesaal round the tables and drink beer *ad lib.* whilst listening to music or in conversation. These entertainments are not always edifying, for I remember one German Herr who boasted of having consumed 16 glasses of beer during the evening.

The patients are examined by the chief doctor only once a month, for the changes in the physical signs are so slow that it is only by examinations at long intervals that appreciable changes can be observed. Naturally, these are occasions of great interest not only to the patient but to his or her neighbours, and form the topic of at least a day's conversation. The leitender Arzt is a very conscientious man, and if the climate is unsuitable for a certain case, he will tell the patient so at once, and recommend him to some other place, usually at a lower altitude. Similarly if a patient is cured he is not kept longer than necessary, but is asked to go in order to make place for someone else. And here I may remark that only slight cases are taken, and if by chance a severe case does arrive, the patient is usually soon sent away again. The cost of living is rather heavy, but not more than in a private sanatorium in England, and is not unreasonable when one remembers that most of the food has to be brought by rail from the plains below. The pension here costs 12 francs a day; besides that one pays for a room according to size and position. A comfortable room facing south costs 5 francs a day. It is possible to live for about £5 10s. a week fairly comfortably. Of course all wines, etc., are extra, so also medicines and any food outside the menu.

A visitor is at once struck with the healthy appearance of the patients, and, when compared with those consumptives who live in pensions or hotels, as very many do, one cannot help noticing a great difference in favour of the former. The people here all seem to make the best of life, and everyone is merry more or less. There is an air of optimism which affects slight and severe cases alike. In the Speisesaal during the meals it is often quite difficult to make oneself heard on account of the chatter and laughing. We often regard ourselves as one large family of which the chief doctor is the father, and, in fact, he generally goes by this name.

The weather in Davos is very variable, and we only get about 170 fine days in the year. At times there is a very strong dry wind from the south called the Föhn. This has the effect of increasing the temperature of most patients, and in consequence, if anyone is not so well he always attributes the cause to the weather. We get a certain amount of snow all the year round, and on July 2 of this year there was quite a heavy snowfall lasting some five or six hours. One day in Davos it is so hot that only flannels are endurable, and the next one must be warmly clad and wear a heavy overcoat. But for all these drawbacks the air is quite pure and always fairly dry, two essentials for Lungenkranke.

Of course a certain amount of nursing is always required here. This is carried out by Sisters of Mercy, who live in a convent close at hand. They are fairly efficient and work very hard, although one can have the exclusive use of a nurse for 2½ francs, *i.e.*, 2s. 1d. a day. They take all their meals in the convent, so one can see at a glance the great difference of cost between having one of these sisters and a trained nurse. Of course, they are not so good as trained nurses, though the majority have had some experience in a clinique. Yet they are much loved by all the patients for their kindness and self-sacrificing nature. One in particular nursed me for five months while I was in bed, and she tended me as a mother would her child; it is owing to her unwearied efforts on my behalf that I am alive at the present. It is interesting to notice how sympathetic the patients are. As soon as a member is missing from the Liegehalle or Speisesaal inquiries are at once made by the other patients, and in my own case if I went to bed for a day I generally had six or seven visitors to see what was the matter, and presents of flowers and books also now and then.

The average length of stay for an early case is about eight months, rarely less. When we consider that the average stay in an English sanatorium is four months we see, perhaps, the reason for so many relapses. Relapsed cases are not common, and this is probably due to the fact that the patient is kept until every trace of disease has vanished.

During the last few weeks of cure patients receive a douche under high pressure every morning, which is at once followed by a long walk involving climbing.

Tuberculin is given to only a certain class of cases, and the results are often astonishingly good. Of course great care is given in the administration of this powerful agent, and largely owing to that this place has gained a world-wide reputation.

Though the above remarks are very disconnected, yet they will give a general idea of the course of life pursued by the patients. The old idea of forced feeding has now expired, and it is now recognised that this is by no means a necessary factor in recovery, but that pure air, light, rest, a hopeful temperament, and a plentiful supply of good food are all equally necessary.

